# Crystal structure of ethyl 5-acetyl-2-{[(di­methyl­amino)­methyl­idene]amino}-4-methyl­thio­phene-3-carboxyl­ate

**DOI:** 10.1107/S2056989015016217

**Published:** 2015-09-17

**Authors:** N. L. Prasad, M. S. Krishnamurthy, H. Nagarajaiah, Noor Shahina Begum

**Affiliations:** a Department of Studies in Chemistry, Central College Campus, Bangalore University, Bangalore 560 001, Karnataka, India

**Keywords:** crystal structure, thio­phene derivative, hydrogen bonding, C—H⋯π inter­actions

## Abstract

In the title thio­phene derivative, C_13_H_18_N_2_O_3_S, the dihedral angles between the thio­phene ring and the [(di­methyl­amino)­methyl­idene]amino side chain (r.m.s. deviation = 0.009 Å) and the –CO_2_ ester group are 3.01 (16) and 59.9 (3)°, respectively. In the crystal, inversion dimers linked by pairs of C—H⋯O hydrogen bonds generate *R*_2_^2^(16) loops. The dimers are linked by another weak C—H⋯O inter­action, forming chains along [001]. In addition, weak C—H⋯π inter­actions are observed, which link the chains into (001) layers.

## Related literature   

For background to the applications of thio­phene derivatives, see: Sabnis *et al.* (1999 ▸). For a related structure, see: Mukhtar *et al.* (2010 ▸). For further synthetic details, see: Gewald *et al.* (1966 ▸).
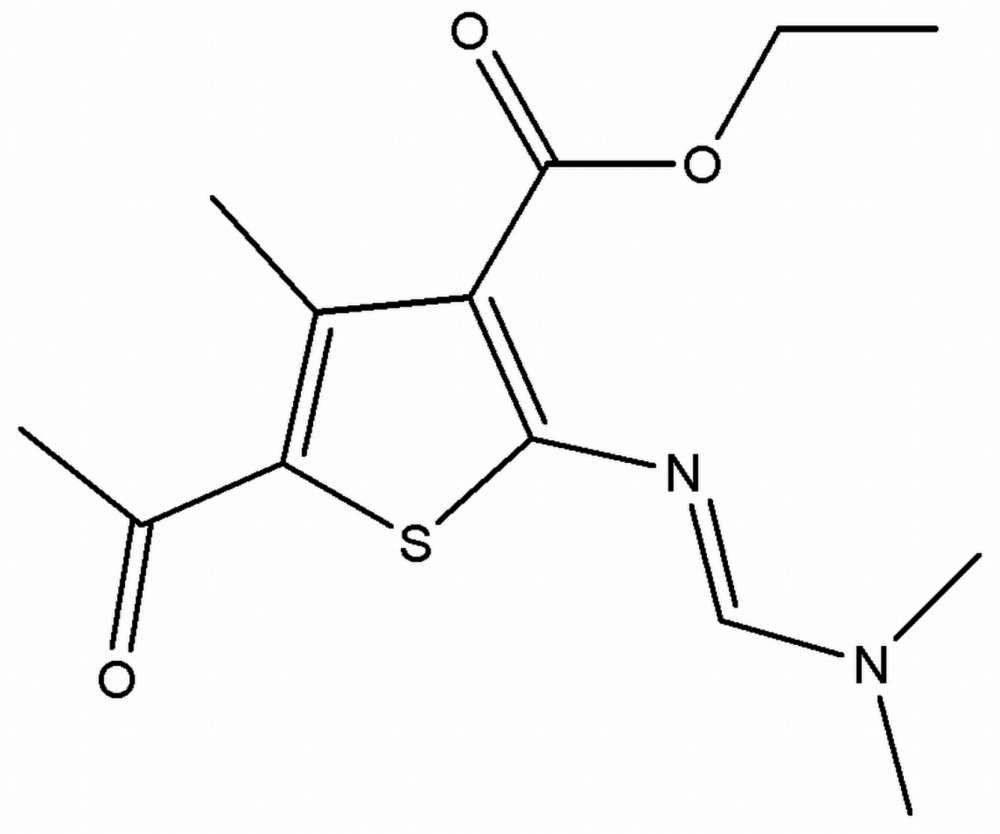


## Experimental   

### Crystal data  

C_13_H_18_N_2_O_3_S
*M* *_r_* = 282.35
Orthorhombic, 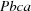

*a* = 12.218 (3) Å
*b* = 7.332 (2) Å
*c* = 30.923 (8) Å
*V* = 2769.9 (13) Å^3^
*Z* = 8
Mo *K*α radiation
μ = 0.24 mm^−1^
*T* = 100 K
0.29 × 0.26 × 0.10 mm

### Data collection  

Bruker SMART APEX CCD diffractometer
Absorption correction: multi-scan (*SADABS*; Bruker, 1998 ▸) *T*_min_ = 0.958, *T*_max_ = 0.963
15478 measured reflections
3012 independent reflections
2140 reflections with *I* > 2σ(*I*)
*R*_int_ = 0.076

### Refinement  

*R*[*F* ^2^ > 2σ(*F* ^2^)] = 0.058
*wR*(*F* ^2^) = 0.173
*S* = 1.19
3012 reflections
177 parameters
H-atom parameters constrained
Δρ_max_ = 0.52 e Å^−3^
Δρ_min_ = −0.34 e Å^−3^

### 

Data collection: *SMART* (Bruker, 1998 ▸); cell refinement: *SAINT-Plus* (Bruker, 1998 ▸); data reduction: *SAINT-Plus*; program(s) used to solve structure: *SHELXS97* (Sheldrick, 2008 ▸); program(s) used to refine structure: *SHELXL97* (Sheldrick, 2008 ▸); molecular graphics: *ORTEP-3 for Windows* (Farrugia, 2012 ▸) and *CAMERON* (Watkin *et al.*, 1996 ▸); software used to prepare material for publication: *WinGX* (Farrugia, 2012 ▸).

## Supplementary Material

Crystal structure: contains datablock(s) global, I. DOI: 10.1107/S2056989015016217/hb7483sup1.cif

Structure factors: contains datablock(s) I. DOI: 10.1107/S2056989015016217/hb7483Isup2.hkl

Supporting information file. DOI: 10.1107/S2056989015016217/hb7483Isup3.cml

**Fig. 1.**
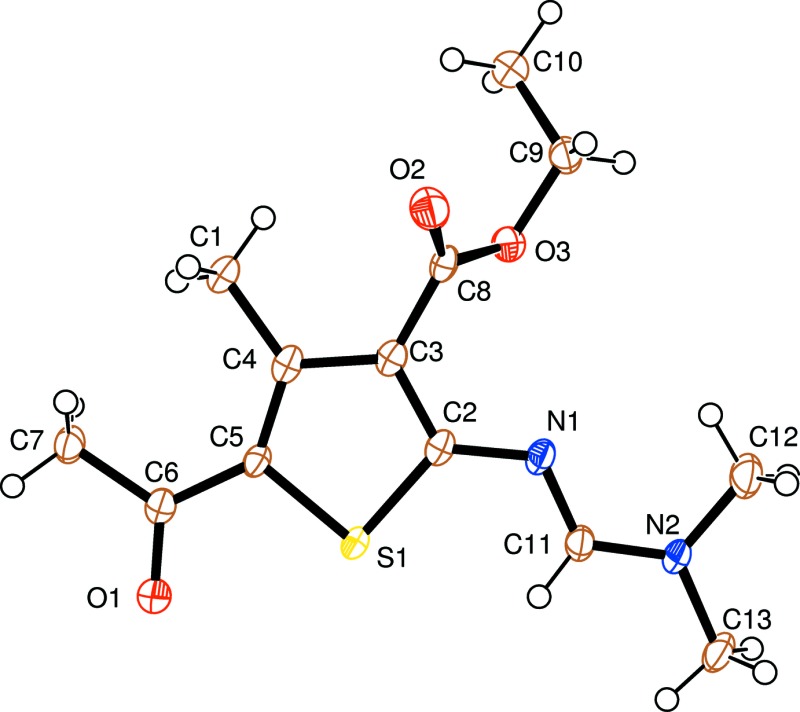
The molecular structure of the title compound with displacement ellipsoids drawn at the 50% probability level. H atoms are presented as small spheres of arbitrary radius.

. DOI: 10.1107/S2056989015016217/hb7483fig1.tif
The mol­ecular structure of the title compound with displacement ellipsoids drawn at the 50% probability level. H atoms are presented as small spheres of arbitrary radius.

**Fig. 2.**
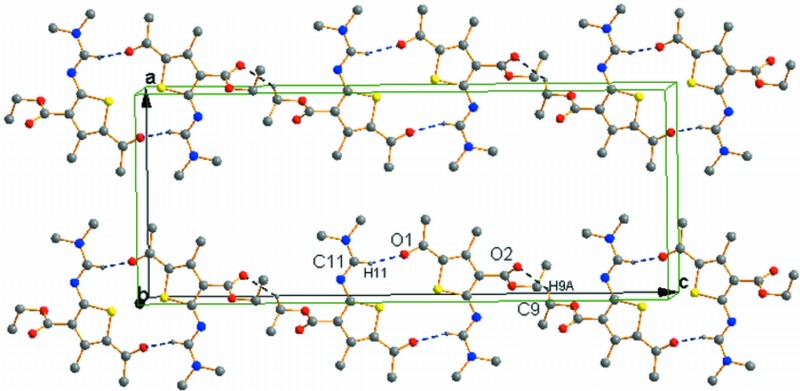
Unit cell packing of the title compound showing intermolecular C—H···O interactions with dotted lines. H-atoms not involved in hydrogen bonding have been excluded.

. DOI: 10.1107/S2056989015016217/hb7483fig2.tif
Unit cell packing of the title compound showing inter­molecular C—H⋯O inter­actions with dotted lines. H-atoms not involved in hydrogen bonding have been excluded.

**Fig. 3.**
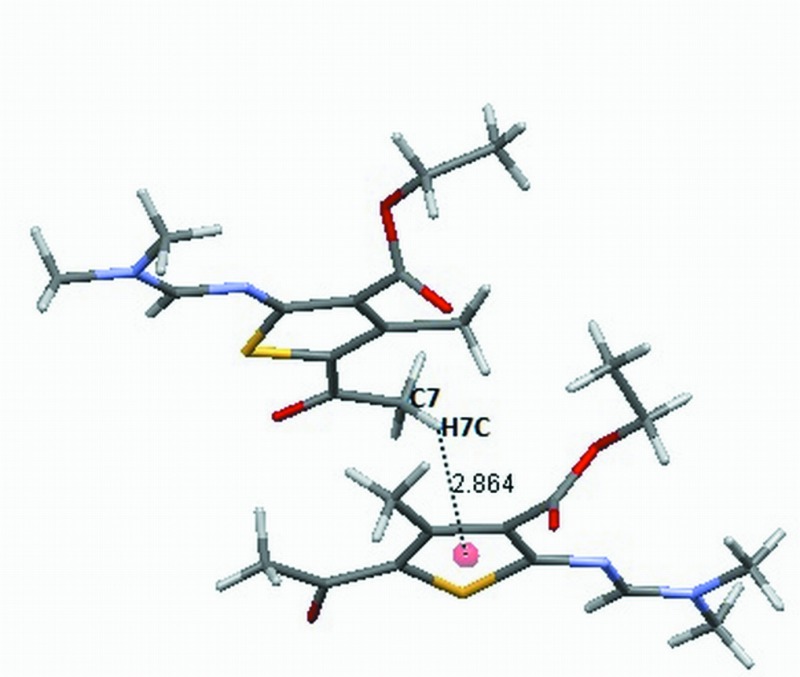
Unit cell packing depicting C—H···π interactions with dotted lines.

. DOI: 10.1107/S2056989015016217/hb7483fig3.tif
Unit cell packing depicting C—H⋯π inter­actions with dotted lines.

CCDC reference: 1421360

Additional supporting information:  crystallographic information; 3D view; checkCIF report

## Figures and Tables

**Table 1:** Hydrogen-bond geometry (, ) *Cg* is the centroid of the C2/C3/C4/C5/S1 ring.

*D*H*A*	*D*H	H*A*	*D* *A*	*D*H*A*
C9H9*A*O2^i^	0.99	2.45	3.270(3)	139
C11H11O1^ii^	0.95	2.47	3.312(4)	147
C7H7*C* *Cg* ^iii^	0.98	2.86	3.693(2)	143

Symmetry codes: (i) 
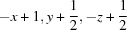
; (ii) 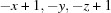; (iii) 
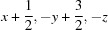
.

## supplementary crystallographic information

### S1. Comment

Thiophene belongs to a class of heterocyclic compounds containing a five membered ring made up of one sulfur as heteroatom, that are widely used as building blocks in many agrochemicals and pharmaceuticals. 2-Aminothiophenes attract special attention because of their applications in pharmaceuticals, agriculture, pesticides and dyes (Sabnis *et al.*, 1999). The most convergent and well established classical approach for the preparation of 2-aminothiophenes is Gewald's method (Gewald *et al.*, 1966), which involves the multicomponent condensation of a ketone with an activated nitrile and elemental sulfur in the presence of diethylamine as a catalyst. Herein, we report the structure of the title compound, (I).

The molecular structure of the compound is shown in Fig. 1. In the title compound, C_13_H_18_N_2_O_3_S, a thiophene derivative with dimethylamino- methyleneamino, acetyl, methyl and ethyl carboxylate substituents attached to a central thiophene ring. The thiophene ring and all the substituents are almost planar except the carboxyl group (C10/C9/O3/C8), it is slightly deviating from the plane at -83.474 (3)°. The carbonyl group of the exocyclic ester at C3 and acetyl at C5 adopts a *trans* orientation with C3=C2 and C5=C4 double bond respectively. The crystal structure features C—H···O interactions. The C11—H11···O1 hydrogen bonds resulting in a centrosymmetric head to head dimer with graph set *R*^2^_2_(16) notation, which are in turn linked by another weak C9—H9A···O2 interactions to form chains of rings along [001] (Table.1; Fig. 2). In addition, weak C—H···π interactions of the type C7—H7C···*Cg* [*Cg* being the centroid of the thiophene ring (C2/C3/C4/C5/S1)] link the chains into layers parallel to (001) with a distance 2.864 Å is also observed (Fig. 3).

### S2. Experimental

Step-1: 3.3 g of cyano ethyl acetate was weighed and transferred to RB flask and 5 g of acetyl acetone and 10 to 15 ml of ethanol were added to it. The whole mixture was stirred for 10 min. After stirring 1.6 g of elemental sulfur was added to the mixture and cold condition was maintained by using crushed ice. Later 5 ml of diethyl amine was added drop by drop the solution changes its color to red. After the completion of addition the solution was again kept for stirring (10 min). Ice pack was removed and stirring was continued for about an hour. The precipitated product (1) was filtered, dried and recrystallized from ethanol (yield: 68%, m.p. 430 K)

Step-2: A mixture of compound 1 (10 mmol) and DMF—DMA (5 ml) was stirred at room temperature for 30 minutes. To this was added ethanol and kept in room temperature to give a solid product (title compound) that was collected by filtration. The compound was recrystallized by slow evaporation from ethanol, yielding single crystals suitable for X-ray diffraction studies.

### S3. Refinement

The H atoms were placed at calculated positions in the riding-model approximation with C—H = 0.96° A, 0.97 ° A and 0.93 ° A for methyl, methylene and methyne H-atoms respectively, with *U*_iso_(H) = 1.5*U*_eq_(C) for methyl H atoms and *U*_iso_(H) = 1.2Ueq(C) for other hydrogen atoms.

### Crystal data

**Table:**

C_13_H_18_N_2_O_3_S	*F*(000) = 1200
*M**_r_* = 282.35	*D*_x_ = 1.354 Mg m^−^^3^
Orthorhombic, *P**b**c**a*	Mo *K*α radiation, λ = 0.71073 Å
Hall symbol: -P 2ac 2ab	Cell parameters from 3012 reflections
*a* = 12.218 (3) Å	θ = 2.1–27.0°
*b* = 7.332 (2) Å	µ = 0.24 mm^−^^1^
*c* = 30.923 (8) Å	*T* = 100 K
*V* = 2769.9 (13) Å^3^	Block, colorless
*Z* = 8	0.29 × 0.26 × 0.10 mm

### Data collection

**Table:**

Bruker SMART APEX CCD diffractometer	3012 independent reflections
Radiation source: fine-focus sealed tube	2140 reflections with *I* > 2σ(*I*)
Graphite monochromator	*R*_int_ = 0.076
ω scans	θ_max_ = 27.0°, θ_min_ = 2.1°
Absorption correction: multi-scan (*SADABS*; Bruker, 1998)	*h* = −14→15
*T*_min_ = 0.958, *T*_max_ = 0.963	*k* = −9→9
15478 measured reflections	*l* = −39→35

### Refinement

**Table:**

Refinement on *F*^2^	Primary atom site location: structure-invariant direct methods
Least-squares matrix: full	Secondary atom site location: difference Fourier map
*R*[*F*^2^ > 2σ(*F*^2^)] = 0.058	Hydrogen site location: inferred from neighbouring sites
*wR*(*F*^2^) = 0.173	H-atom parameters constrained
*S* = 1.19	*w* = 1/[σ^2^(*F*_o_^2^) + (0.0811*P*)^2^ + 0.3374*P*] where *P* = (*F*_o_^2^ + 2*F*_c_^2^)/3
3012 reflections	(Δ/σ)_max_ < 0.001
177 parameters	Δρ_max_ = 0.52 e Å^−^^3^
0 restraints	Δρ_min_ = −0.33 e Å^−^^3^

### Special details

**Table:**

Geometry. All s.u.'s (except the s.u. in the dihedral angle between two l.s. planes) are estimated using the full covariance matrix. The cell s.u.'s are taken into account individually in the estimation of s.u.'s in distances, angles and torsion angles; correlations between s.u.'s in cell parameters are only used when they are defined by crystal symmetry. An approximate (isotropic) treatment of cell s.u.'s is used for estimating s.u.'s involving l.s. planes.
Refinement. Refinement of *F*^2^ against ALL reflections. The weighted *R*-factor *wR* and goodness of fit *S* are based on *F*^2^, conventional *R*-factors *R* are based on *F*, with *F* set to zero for negative *F*^2^. The threshold expression of *F*^2^ > 2σ(*F*^2^) is used only for calculating *R*-factors(gt) *etc*. and is not relevant to the choice of reflections for refinement. *R*-factors based on *F*^2^ are statistically about twice as large as those based on *F*, and *R*- factors based on ALL data will be even larger.

### Fractional atomic coordinates and isotropic or equivalent isotropic displacement parameters (Å^2^)

**Table:**

	*x*	*y*	*z*	*U*_iso_*/*U*_eq_	
C1	0.8067 (2)	0.2414 (4)	0.37436 (9)	0.0219 (6)	
H1A	0.8527	0.3124	0.3942	0.033*	
H1B	0.7973	0.3089	0.3473	0.033*	
H1C	0.8419	0.1240	0.3684	0.033*	
C2	0.5031 (2)	0.1973 (4)	0.39447 (9)	0.0194 (6)	
C3	0.5979 (2)	0.2312 (4)	0.37104 (9)	0.0193 (6)	
C4	0.6967 (2)	0.2091 (4)	0.39477 (9)	0.0196 (6)	
C5	0.6764 (2)	0.1574 (4)	0.43709 (9)	0.0190 (6)	
C6	0.7476 (2)	0.1108 (4)	0.47350 (9)	0.0211 (6)	
C7	0.8697 (2)	0.1198 (4)	0.46853 (9)	0.0252 (7)	
H7A	0.9048	0.0694	0.4944	0.038*	
H7B	0.8923	0.2471	0.4648	0.038*	
H7C	0.8919	0.0488	0.4431	0.038*	
C8	0.5956 (2)	0.2660 (4)	0.32370 (9)	0.0203 (6)	
C9	0.5315 (2)	0.4612 (4)	0.26762 (9)	0.0251 (7)	
H9A	0.4661	0.5384	0.2628	0.030*	
H9B	0.5215	0.3467	0.2511	0.030*	
C10	0.6313 (2)	0.5590 (5)	0.25110 (10)	0.0321 (7)	
H10A	0.6411	0.6730	0.2672	0.048*	
H10B	0.6219	0.5864	0.2203	0.048*	
H10C	0.6958	0.4814	0.2550	0.048*	
C11	0.3149 (2)	0.1822 (4)	0.40202 (9)	0.0216 (6)	
H11	0.3262	0.1544	0.4317	0.026*	
C13	0.1194 (2)	0.1650 (4)	0.41533 (10)	0.0287 (7)	
H13A	0.1452	0.1305	0.4442	0.043*	
H13B	0.0736	0.0673	0.4035	0.043*	
H13C	0.0765	0.2776	0.4173	0.043*	
C12	0.1888 (2)	0.2409 (5)	0.34251 (10)	0.0323 (8)	
H12A	0.2567	0.2747	0.3277	0.048*	
H12B	0.1377	0.3439	0.3418	0.048*	
H12C	0.1558	0.1356	0.3280	0.048*	
N1	0.40009 (18)	0.2074 (3)	0.37699 (7)	0.0216 (5)	
N2	0.21291 (18)	0.1943 (3)	0.38709 (7)	0.0218 (5)	
O1	0.70690 (16)	0.0608 (3)	0.50809 (6)	0.0285 (5)	
O2	0.63937 (18)	0.1686 (3)	0.29724 (6)	0.0308 (5)	
O3	0.54047 (16)	0.4187 (3)	0.31356 (6)	0.0244 (5)	
S1	0.53616 (5)	0.13387 (10)	0.44751 (2)	0.0202 (2)	

### Atomic displacement parameters (Å^2^)

**Table:**

	*U*^11^	*U*^22^	*U*^33^	*U*^12^	*U*^13^	*U*^23^
C1	0.0151 (15)	0.0206 (15)	0.0301 (16)	−0.0008 (11)	0.0015 (11)	−0.0017 (12)
C2	0.0147 (14)	0.0169 (14)	0.0267 (15)	0.0005 (11)	−0.0003 (11)	−0.0026 (11)
C3	0.0166 (15)	0.0142 (14)	0.0270 (15)	0.0008 (10)	0.0002 (11)	−0.0016 (11)
C4	0.0154 (15)	0.0127 (14)	0.0308 (15)	−0.0002 (10)	−0.0009 (11)	−0.0039 (11)
C5	0.0125 (14)	0.0223 (15)	0.0220 (14)	0.0018 (11)	0.0012 (11)	−0.0017 (11)
C6	0.0176 (15)	0.0189 (15)	0.0268 (15)	0.0001 (11)	−0.0015 (12)	−0.0001 (11)
C7	0.0170 (15)	0.0281 (16)	0.0304 (16)	−0.0015 (12)	−0.0037 (12)	0.0031 (12)
C8	0.0133 (14)	0.0197 (15)	0.0278 (16)	−0.0041 (11)	−0.0025 (12)	−0.0015 (12)
C9	0.0231 (16)	0.0261 (16)	0.0262 (15)	−0.0016 (12)	−0.0041 (12)	0.0043 (12)
C10	0.0262 (17)	0.0424 (19)	0.0276 (16)	−0.0008 (14)	−0.0002 (14)	0.0053 (14)
C11	0.0165 (15)	0.0236 (15)	0.0248 (15)	0.0010 (11)	−0.0026 (12)	0.0007 (12)
C13	0.0131 (15)	0.0363 (18)	0.0368 (18)	−0.0005 (12)	0.0015 (13)	0.0009 (14)
C12	0.0196 (16)	0.043 (2)	0.0341 (18)	0.0057 (13)	−0.0040 (13)	−0.0024 (15)
N1	0.0140 (13)	0.0235 (13)	0.0275 (13)	0.0013 (10)	−0.0003 (10)	0.0009 (10)
N2	0.0130 (12)	0.0285 (14)	0.0240 (12)	0.0006 (10)	0.0000 (10)	0.0005 (10)
O1	0.0216 (11)	0.0385 (13)	0.0254 (11)	−0.0008 (9)	−0.0010 (9)	0.0059 (9)
O2	0.0307 (12)	0.0372 (13)	0.0245 (11)	0.0088 (10)	−0.0012 (9)	−0.0060 (9)
O3	0.0225 (11)	0.0257 (11)	0.0251 (11)	0.0029 (8)	0.0001 (8)	0.0028 (9)
S1	0.0121 (4)	0.0250 (4)	0.0237 (4)	−0.0003 (3)	0.0004 (3)	0.0017 (3)

### Geometric parameters (Å, º)

**Table:**

C1—C4	1.504 (4)	C9—O3	1.458 (3)
C1—H1A	0.9800	C9—C10	1.504 (4)
C1—H1B	0.9800	C9—H9A	0.9900
C1—H1C	0.9800	C9—H9B	0.9900
C2—N1	1.372 (3)	C10—H10A	0.9800
C2—C3	1.388 (4)	C10—H10B	0.9800
C2—S1	1.752 (3)	C10—H10C	0.9800
C3—C4	1.422 (4)	C11—N1	1.310 (3)
C3—C8	1.486 (4)	C11—N2	1.332 (3)
C4—C5	1.385 (4)	C11—H11	0.9500
C5—C6	1.464 (4)	C13—N2	1.454 (4)
C5—S1	1.752 (3)	C13—H13A	0.9800
C6—O1	1.235 (3)	C13—H13B	0.9800
C6—C7	1.501 (4)	C13—H13C	0.9800
C7—H7A	0.9800	C12—N2	1.450 (4)
C7—H7B	0.9800	C12—H12A	0.9800
C7—H7C	0.9800	C12—H12B	0.9800
C8—O2	1.210 (3)	C12—H12C	0.9800
C8—O3	1.343 (3)		
			
C4—C1—H1A	109.5	C10—C9—H9A	109.2
C4—C1—H1B	109.5	O3—C9—H9B	109.2
H1A—C1—H1B	109.5	C10—C9—H9B	109.2
C4—C1—H1C	109.5	H9A—C9—H9B	107.9
H1A—C1—H1C	109.5	C9—C10—H10A	109.5
H1B—C1—H1C	109.5	C9—C10—H10B	109.5
N1—C2—C3	123.4 (3)	H10A—C10—H10B	109.5
N1—C2—S1	126.5 (2)	C9—C10—H10C	109.5
C3—C2—S1	110.1 (2)	H10A—C10—H10C	109.5
C2—C3—C4	114.7 (3)	H10B—C10—H10C	109.5
C2—C3—C8	122.0 (2)	N1—C11—N2	121.9 (3)
C4—C3—C8	122.9 (2)	N1—C11—H11	119.0
C5—C4—C3	111.5 (2)	N2—C11—H11	119.0
C5—C4—C1	126.8 (2)	N2—C13—H13A	109.5
C3—C4—C1	121.6 (3)	N2—C13—H13B	109.5
C4—C5—C6	133.2 (3)	H13A—C13—H13B	109.5
C4—C5—S1	112.1 (2)	N2—C13—H13C	109.5
C6—C5—S1	114.7 (2)	H13A—C13—H13C	109.5
O1—C6—C5	119.7 (3)	H13B—C13—H13C	109.5
O1—C6—C7	120.2 (2)	N2—C12—H12A	109.5
C5—C6—C7	120.1 (2)	N2—C12—H12B	109.5
C6—C7—H7A	109.5	H12A—C12—H12B	109.5
C6—C7—H7B	109.5	N2—C12—H12C	109.5
H7A—C7—H7B	109.5	H12A—C12—H12C	109.5
C6—C7—H7C	109.5	H12B—C12—H12C	109.5
H7A—C7—H7C	109.5	C11—N1—C2	119.2 (2)
H7B—C7—H7C	109.5	C11—N2—C12	122.3 (2)
O2—C8—O3	123.7 (3)	C11—N2—C13	121.1 (2)
O2—C8—C3	123.8 (3)	C12—N2—C13	116.5 (2)
O3—C8—C3	112.5 (2)	C8—O3—C9	116.3 (2)
O3—C9—C10	111.8 (2)	C2—S1—C5	91.56 (13)
O3—C9—H9A	109.2		
			
N1—C2—C3—C4	−178.5 (2)	C2—C3—C8—O2	−117.1 (3)
S1—C2—C3—C4	−0.6 (3)	C4—C3—C8—O2	55.5 (4)
N1—C2—C3—C8	−5.4 (4)	C2—C3—C8—O3	63.5 (3)
S1—C2—C3—C8	172.5 (2)	C4—C3—C8—O3	−124.0 (3)
C2—C3—C4—C5	0.0 (3)	N2—C11—N1—C2	178.5 (2)
C8—C3—C4—C5	−173.0 (2)	C3—C2—N1—C11	−176.6 (3)
C2—C3—C4—C1	−179.7 (2)	S1—C2—N1—C11	5.8 (4)
C8—C3—C4—C1	7.3 (4)	N1—C11—N2—C12	−1.7 (4)
C3—C4—C5—C6	177.1 (3)	N1—C11—N2—C13	179.7 (3)
C1—C4—C5—C6	−3.2 (5)	O2—C8—O3—C9	2.1 (4)
C3—C4—C5—S1	0.6 (3)	C3—C8—O3—C9	−178.4 (2)
C1—C4—C5—S1	−179.8 (2)	C10—C9—O3—C8	−83.4 (3)
C4—C5—C6—O1	−176.9 (3)	N1—C2—S1—C5	178.6 (2)
S1—C5—C6—O1	−0.4 (3)	C3—C2—S1—C5	0.7 (2)
C4—C5—C6—C7	1.8 (5)	C4—C5—S1—C2	−0.8 (2)
S1—C5—C6—C7	178.2 (2)	C6—C5—S1—C2	−178.0 (2)

### Hydrogen-bond geometry (Å, º)

Cg is the centroid of the C2/C3/C4/C5/S1 ring .

**Table:**

*D*—H···*A*	*D*—H	H···*A*	*D*···*A*	*D*—H···*A*
C9—H9*A*···O2^i^	0.99	2.45	3.270 (3)	139
C11—H11···O1^ii^	0.95	2.47	3.312 (4)	147
C7—H7*C*···*Cg*^iii^	0.98	2.86	3.693 (2)	143

Symmetry codes: (i) −*x*+1, *y*+1/2, −*z*+1/2; (ii) −*x*+1, −*y*, −*z*+1; (iii) *x*+1/2, −*y*+3/2, −*z*.
