# Supplementary figures and images for: Crystal structure of ethyl 5-acetyl-2-{[(di­methyl­amino)­methyl­idene]amino}-4-methyl­thio­phene-3-carboxyl­ate
Source: Acta Crystallogr E Crystallogr Commun. 2015 Sep 17;71(Pt 10):o762–3. doi: 10.1107/S2056989015016217 (PMC4647409; doi:10.1107/S2056989015016217)

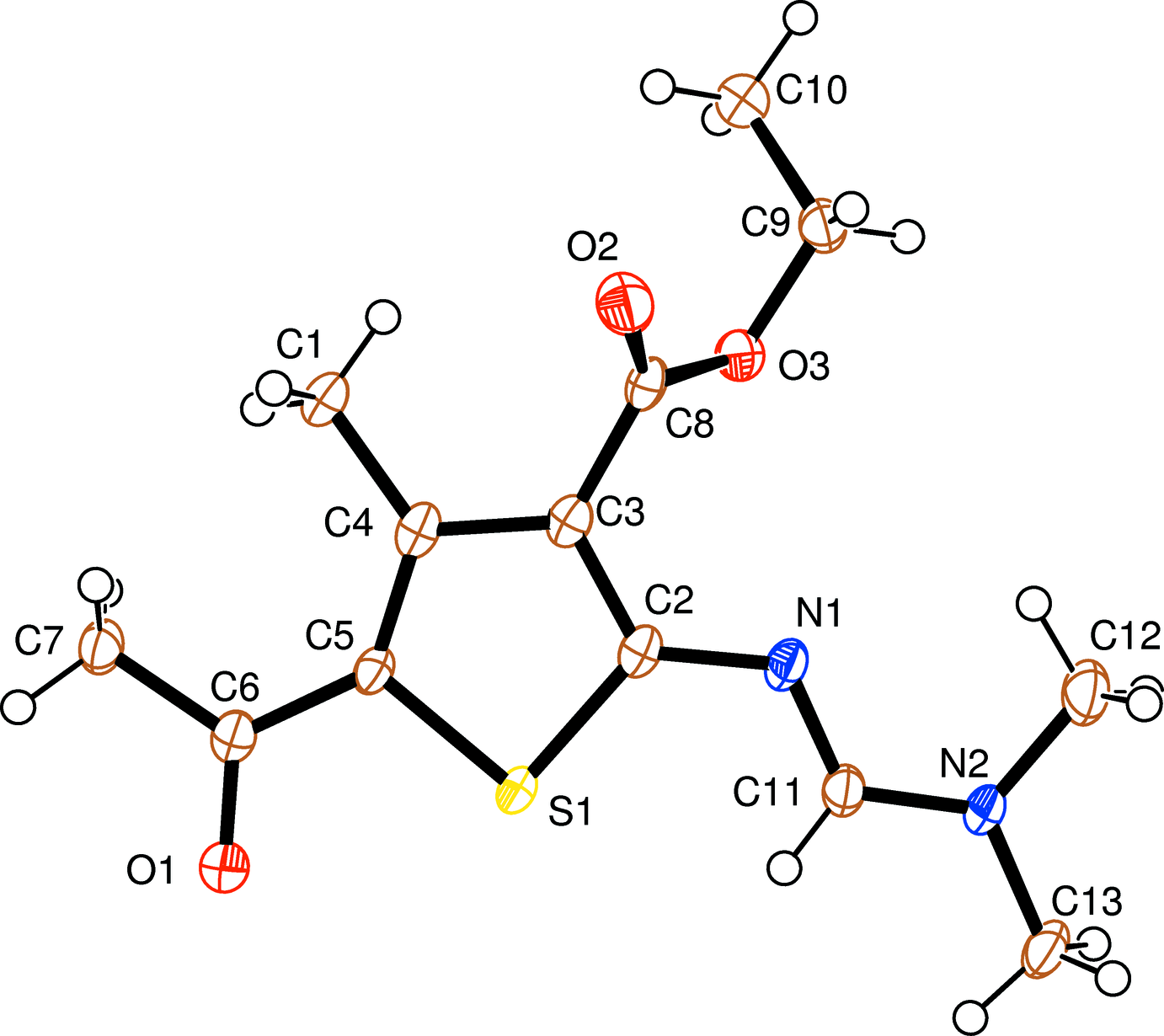

Supplement: Supplementary file 4 [file e-71-0o762-fig1.tif]

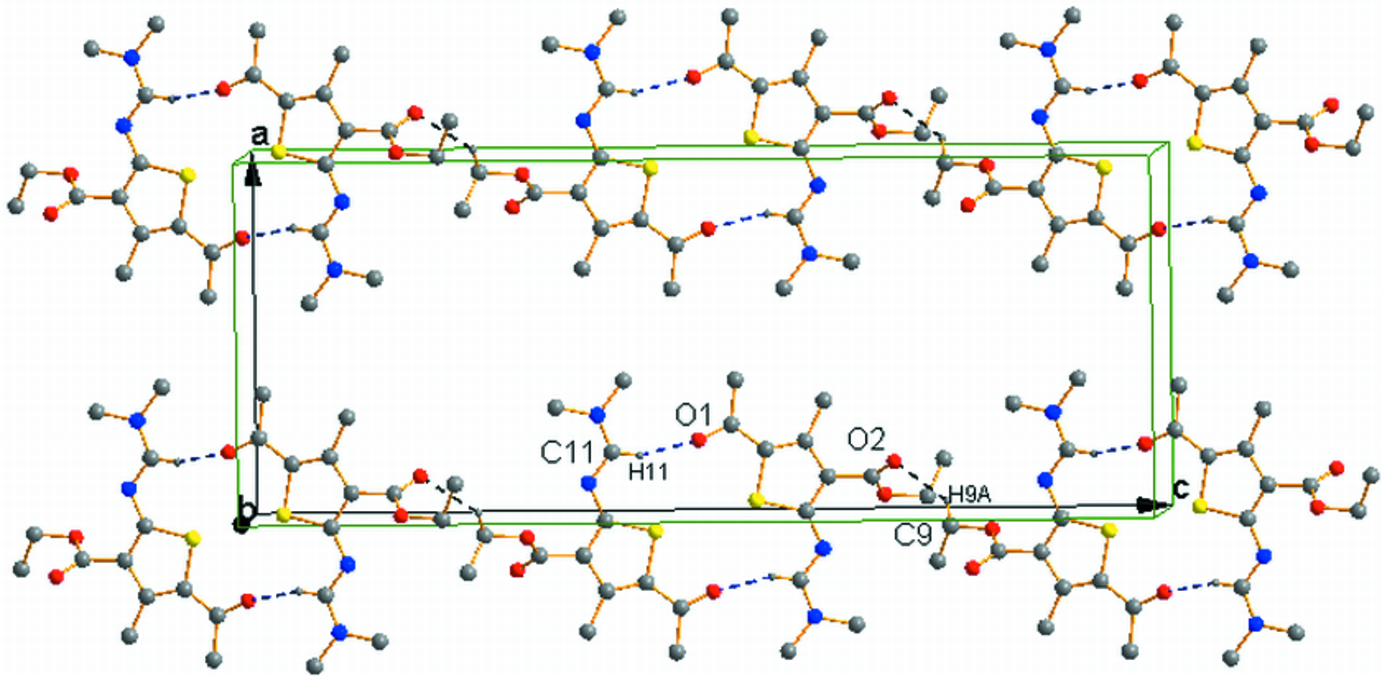

Supplement: Supplementary file 5 [file e-71-0o762-fig2.tif]

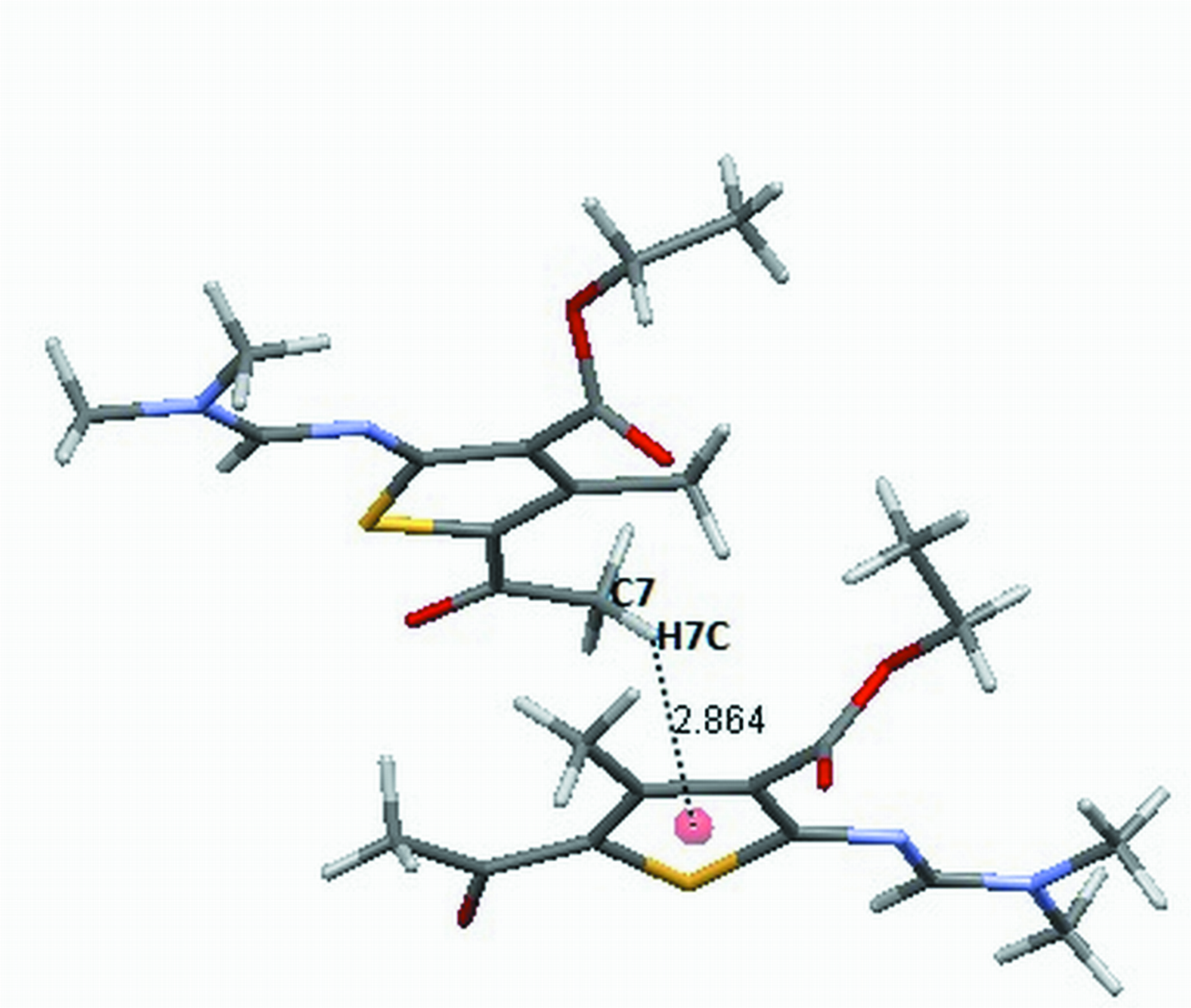

Supplement: Supplementary file 6 [file e-71-0o762-fig3.tif]
